# Effect of Sub-Inhibitory Concentrations of Quaternary Ammonium Compounds and Heavy Metals on Antibiotic Resistance and Expression of Virulence Factors Among *Staphylococcus* spp. from Dairy Products

**DOI:** 10.3390/ijms26062429

**Published:** 2025-03-08

**Authors:** Zuzanna Byczkowska-Rostkowska, Joanna Gajewska, Anna Zadernowska, Wioleta Chajęcka-Wierzchowska

**Affiliations:** Department of Food Microbiology, Meat Technology and Chemistry, Faculty of Food Science, University of Warmia and Mazury in Olsztyn, Plac Cieszyński 1, 10-726 Olsztyn, Poland; zuzanna.byczkowska@uwm.edu.pl (Z.B.-R.); anna.zadernowska@uwm.edu.pl (A.Z.); wioleta.chajecka@uwm.edu.pl (W.C.-W.)

**Keywords:** *Staphylococcus*, gene expression, disinfectants, antimicrobial resistance, biofilm

## Abstract

Antimicrobial resistance is spreading rapidly throughout the world. The food chain can be one of the routes of transmission for microorganisms containing drug-resistance genes and thus serve as a channel for their transmission. Environmental stress and methods of preventing the spread of microorganisms trigger adaptive responses in bacterial cells. The aim of the present study was to determine the effect of the stress induced by sub-inhibitory concentrations (SICs) of cadmium chloride and benzalkonium chloride on antibiotic resistance and the expression of selected virulence factors in *Staphylococcus* isolates from food. The study was conducted on strains of the species *S. epidermidis, S. heamolyticus, S. saprophyticus*, and *S. aureus*. The values of the minimum inhibitory concentration against erythromycin, tetracycline, and oxacillin were determined before and after the incubation of the tested strains under stress conditions. The ability to form biofilm and slime production was also investigated. The expression levels of the genes responsible for antibiotic resistance (*bla*Z, *tet*K, *tet*M, *erm*B, and *mec*A) and virulence (*eno*) were conducted using Real-Time PCR. The MIC values of the antibiotics tested against the strains analyzed were found to be elevated in the presence of SICs of benzalkonium chloride and cadmium chloride. Furthermore, the intensity of biofilm production was also increased. SICs of benzalkonium chloride induced the expression of the *tet*M, *tet*K, *mec*A, and *bla*Z genes in 75%, 66.6%, 33.3%, and 40% of the isolates tested, respectively. Similar treatment with cadmium chloride induced the expression of the same genes in 75%, 100%, 66.6%, and 40% of the strains. In both cases, the expression of the *erm*B gene was reduced in 100% of the isolates. The *eno* gene was found to be overexpressed in 66.6% of the strains following benzalkonium chloride stress, and in 100% of the strains following cadmium chloride stress. These findings suggest that in *Staphylococcus* spp. strains, changes in the expression of the genes encoding antibiotic resistance and virulence factors may occur in response to the applied stress factors. The results indicate the possibility of selecting more resistant and virulent strains due to the use of too low concentrations of disinfectants, which emphasizes the need to use appropriate inhibitory doses of disinfectants in the food industry.

## 1. Introduction

Staphylococci are widespread microorganisms, but not all of them carry virulence factors or exhibit multi-resistance. However, those that do pose a significant concern for public health and food safety [1]. Depending on the presence of virulence factors such as hemolysins and biofilm formation, they can cause a wide range of diseases in animals and humans. Both coagulase-positive staphylococci (CPS) and coagulase-negative staphylococci (CoNS) are the major causes of bovine mastitis [2,3]. Staphylococcal enterotoxins are the most common cause of food poisoning worldwide [3,4]. Species from CPS and CoNS groups have the capacity to produce enterotoxins, thereby underscoring their involvement in potential food poisoning incidents [5].

Foodborne microorganisms are typically exposed to multiple stress factors throughout their life cycle, from farm to table, during food production and processing, which can lead bacteria to sublethal damage or a condition where they are viable but non-culturable (VBNC). Such conditions can be physical stresses such as irradiation, pressure, heat, or osmotic shock, as well as food processing techniques and chemical treatments [6,7].

The use of disinfectants is an effective method of preventing the spread of unwanted microorganisms carried in hospitals, food industry facilities, and households [8]. To prevent the spread of harmful microorganisms, the following are used: disinfectants, such as quaternary ammonium compounds (QACs). The inappropriate use of biocides has been demonstrated to expose microbial life to biocidal agents, whether in the form of low concentrations [9,10] or heavy metal compounds [11].

The dilution and biocide residue phenomenon within environmental systems has been shown to give rise to a concentration gradient of these compounds [12]. As a consequence of this phenomenon, microorganisms are exposed to ineffective concentrations of disinfectants, i.e., non-inhibitory, sub-inhibitory, and post-inhibitory, which causes selection pressure, and thus may result in the development of microbial resistance to a given disinfectant and cross-resistance to other agents, e.g., antibiotics. This can be a temporary or permanent effect resulting from temporary phenotypic adaptations or the selection of stable gene mutations [10]. The presence of biocides in the microbial community at concentrations lower than the MIC (minimum inhibitory concentration) induces various microbial actions to increase resistance to harmful compounds, such as increasing the activity of the efflux pump [10], intensifying biofilm production [13], or translocating resistance genes [14].

QACs, including benzalkonium chloride, are used for teat disinfection to prevent mastitis, which is mainly caused by CoNS [12]. QACs undergo biodegradation under aerobic conditions, which influences their concentrations in the environment to vary. As a result, microorganisms are exposed to QACs over a wide range of concentrations. Sub-inhibitory concentrations of QAC present in the environment are selective for the microorganisms present. This results in the emergence and spread of QAC resistance among various types of bacteria, including clinically relevant pathogens. Resistance pathways to quaternary ammonium compounds and antibiotics appear to be similar, so understanding resistance and the spread of QACs is crucial in the context of the global problem of antibiotic resistance [9].

Heavy metals are called elements whose density is greater than 5 g/cm^3^. They are widespread in the environment and in the cells of living organisms. Compounds that do not have any physiological functions and exhibit strong toxicity are used as disinfectants. These include lead, arsenic, silver, and cadmium [15]. Cadmium and its chemical compounds are used in the food sector for their toxic and disinfectant properties, which result from the disruption of cellular respiration [11]. In agriculture, it is a component of fertilizers, which directly affects its presence in plants that are raw materials for food production [16]. Research conducted on environmental samples has demonstrated that repeated exposure of bacteria to cadmium has been shown to enhance biofilm formation and their resistance to heavy metals [17,18]. Furthermore, the inappropriate use of heavy metal agents, among other things in agricultural production, has been demonstrated to affect bacterial resistance to antibiotics [19].

The presence of bacterial biofilms is a significant factor in the survival of bacteria within the food chain, with the potential to contribute to the contamination of food. Previous studies have demonstrated that benzalkonium chloride, at non-inhibitory concentrations (below the MIC), can enhance the biofilm-forming capacity of strains demonstrating resistance to this disinfectant [20,21].

Due to the fact that the spread of antibiotic resistance is currently one of the major concerns, there has been a need to intensify efforts to understand the impact of biocide and heavy metals on antibiotic resistance and the virulence of bacteria. Therefore, the aim of our study was to determine the effect of sub-inhibitory concentrations (SICs) of disinfectants with QAC (based on benzalkonium chloride) and heavy metals compounds (based on cadmium chloride) on antibiotic resistance and the expression of virulence factors in strains of the genus *Staphylococcus* from food.

## 2. Results

### 2.1. Susceptibility of Strains to Benzalkonium and Cadmium Chloride

Significantly reduced sensitivity to benzalkonium chloride was found in six strains (18.8%), in which the minimum inhibitory concentration was at least 8 μg/mL. Due to the lack of standardized and unified cut-off values, we classified 22 (68.8%) strains (MIC ≥ 4 μg/mL) among those with partially reduced sensitivity. Among the *S. aureus* strains, reduced sensitivity to benzalkonium chloride was exhibited by 100% of the strains (MIC = 4 μg/mL). In contrast, among CoNS, it was 85.7%, of which six strains had MICs higher than 4 μg/mL. The highest MIC value was 16 μg/mL and was recorded in one strain belonging to the *S. epidermidis* species. Other strains distinguished by significantly reduced sensitivity belonged to the species *S. epidermidis* and *S. haemolyticus.* (MIC = 8 μg/mL). The lowest recorded MIC value was 2 μg/mL, which was detected in four of the tested strains (12.5%)—all belonged to the group of CoNS.

The values of MIC of cadmium chloride significantly exceeded those recorded for benzalkonium chloride. In our research, with cadmium chloride, as many as 26 strains (81.3%) had MICs ≥ 16 μg/mL. The highest MIC, contributing 256 μg/mL, was observed among two *S. aureus* strains (6.3%). Four other CPS-tested strains had a reduced sensitivity of 128 μg/mL. In the case of CoNS, on the other hand, the value of 128 μg/mL remained the highest recorded MIC, indicating significantly reduced sensitivity of two strains belonging to the species *S. epidermidis* and *S. saprophyticus*. Among the remaining CoNS, the most commonly recorded MIC value was 16 μg/mL (in 42.6%). Lower MIC values of 0.25, 0.5, and 4 μg/mL, which were not recorded for *S. aureus*, also appeared in this group. Also worth noting was strain 14G, which belongs to the *S. epidermidis* species. It showed relatively strongly reduced sensitivity to both benzalkonium chloride (MIC = 8 μg/mL) and cadmium chloride (MIC = 128 μg/mL) (Table 1).

An increase in the MIC values after incubation in the presence of SIC of benzalkonium chloride, and thus a reduction in sensitivity to this disinfectant, was observed in 68.8% (n = 22) of the tested strains. In 25% (n = 8) of the strains, the MIC value was the same as before the study. However, in two strains (6.3%) a decrease in MIC value was observed after exposure to SIC.

A two-fold increase was the most common, occurring in 56.3% of the strains. On the other hand, four-fold growth affected 12.5% of strains. In both CoNS and CPS, a two-fold increase in the MIC values was most often observed (57.1% and 85.7%, respectively).

Incubation in the presence of SIC of cadmium chloride resulted in an increase in the MIC values in 62.5% (n = 20) of the strains, so a reduction in sensitivity to this disinfectant occurred in fewer strains than with benzalkonium chloride. The minimum inhibitory concentration equal to the pre-test value was maintained in 9.4% (n = 3) of the isolates, while its decrease affected 28.1% (n = 9) of the strains. The most frequently observed increases in the MIC values were two-fold, four-fold, and eight-fold, which affected 12.5% of the strains each. Sixteen-fold increases were also observed in 9.4% of the isolates (n = 3), thirty-two-fold increases in 6.3% of the isolates (n = 2), and sixty-four-fold increases in one 10G isolate (3.1%). In the group of CoNS the *S. epidermidis* species, there were increases in more than two hundred-fold (strain 8G) and more than five hundred-fold (strain 17G).

### 2.2. Analysis of Phenotypic Changes in Response to Sub-Inhibitory Treatment

#### 2.2.1. Antibiotic Susceptibility Changes

An increase in MIC for erythromycin was shown among five (83.3%) of the six isolates after exposure to benzalkonium chloride (no changes for 1G *S. epidermidis*) and for all the isolates after cadmium chloride. It is noteworthy that in two isolates belonging to the species *S. epidermidis* (2G, 3G), the increase in the MIC values changed the interpretation of the result from “intermediate-sensitive” to “resistant” and from “sensitive” to “intermediate-sensitive”. Based on statistical analysis, there was no statistical significance of the difference between the MIC values before and after the stresses (benzalkonium chloride *p* = 0.0644; cadmium chloride *p* = 0.2104) (Table 2).

For tetracycline, the change in the MIC values after the benzalkonium chloride treatment was observed for four isolates tested (*S. epidermidis* 2G and 3G and *S. aureus* 25G and 32G), while no change was observed after the cadmium chloride treatment only for *S. aureus* 24G. The changes in MIC values did not affect the interpretation of the result of the sensitivity of the tested strains to tetracycline despite the statistical significance of the differences for cadmium chloride (*p* = 0.0292). For benzalkonium chloride, there were no statistically significant differences (*p* = 0.0781) (Table 2).

An increase in the MIC values for oxacillin was observed for all the tested strains after exposure of the tested isolates to SICs of benzalkonium chloride. Cadmium chloride stress caused an increase in the MIC values in 5/6 of the tested isolates. Significantly, for one *S. aureus* strain (25G), the impact of stress factors at SICs caused a significant change in the MIC values (from 2 to 16 μg/mL). In the case of strain 25G (*S. aureus*), changes in the MIC values due to cadmium and benzalkonium chloride stress changed the interpretation of sensitivity from susceptible to resistant. Based on statistical analysis, there were no statistically significant differences for benzalkonium chloride (*p* = 0.0639) and cadmium chloride (*p* = 0.1141) (Table 2).

#### 2.2.2. Changes in Ability to Slime Production and Biofilm Formation

The results of our study showed that the 3G strain under stress with SICs of cadmium chloride changed its colony color from “bordeaux” to “almost black”, thus changing its properties from incapable of producing slime to capable of producing slime. For *S. epidermidis* strains (1G and 2G) and *S. aureus* (24G, 25G, and 32G), no color changes in colonies were observed under the applied stress factors. This implies the absence of phenotypic changes concerning the slime-producing ability of these strains.

The applied stress factors had different effects on the intensity of biofilm generation by the tested strains. In strain 1G (*S. epidermidis*), there was a change in the interpretation of biofilm production from moderate to weak because of cadmium chloride. Strain 2G (*S. epidermidis*) changed the intensity of biofilm production from moderate to strong after the effect of both the applied disinfectants at SICs. For strain 3G (*S. epidermidis*), a change in the intensity of biofilm production from weak to moderate was observed after benzalkonium chloride stress. A change in phenotype to strong was noted after cadmium chloride stress. The strains 24G and 25G (*S. aureus*) showed a change in the intensity of biofilm production after sub-inhibitory benzalkonium chloride stress from strong to moderate. For strain 32G (*S. aureus*), no phenotypic changes in biofilm production were observed (Table 3). Based on statistical analysis, there were no statistically significant differences between the OD value for the control strains and the OD value for the strains subjected to the stress factors tested (*p* > 0.05).

### 2.3. Gene Expression Analysis

**Figure 1:**
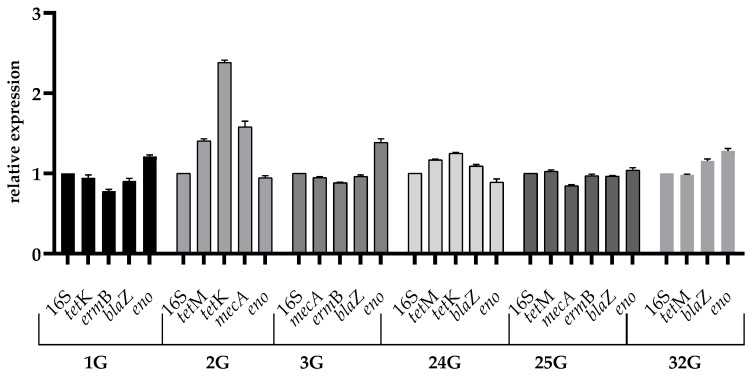
Relative expression of the virulence genes among the tested strains cultured in the sub-inhibitory concentration of benzalkonium chloride. The error bars represent SD.

The sub-inhibitory concentration of benzalkonium chloride contributed to the overexpression of the *tet*M (1.16), *tet*K (1.26), and *bla*Z (1.07) genes of strain 24G (*S. aureus*) and significant overexpression of the *tet*M (1.43), *tet*K (2.41), and *mec*A (1.51) genes of strain 2G (*S. epidermidis*). Increased expression of the *bla*Z gene (1.13) was also observed in strain 32G (*S. aureus*). Decreased expression was noted for the *tet*K (0.91), *erm*B (0.80), and *bla*Z (0.94) genes of strain 1G (*S. epidermidis*). Strain 3G (*S. epidermidis*) showed reduced expression of the *erm*B (0.89) and *mec*A (0.94) genes. Reduced expression of the *mec*A gene was also shown by strain 25G (*S. aureus*). The other genes showed expressions similar to that of the reference gene. The SICs of benzalkonium chloride contributed to the increased expression of the *eno* gene of the strains 1G (1.19) and 3G (1.34) of *S. epidermidis* and 25G (1.07) and 32G (1.25) of *S. aureus*. Decreased values were noted for the *eno* gene of strain 2G (*S. epidermidis*) (0.97) and strain 24G (0.93) (Figure 1).

**Figure 2:**
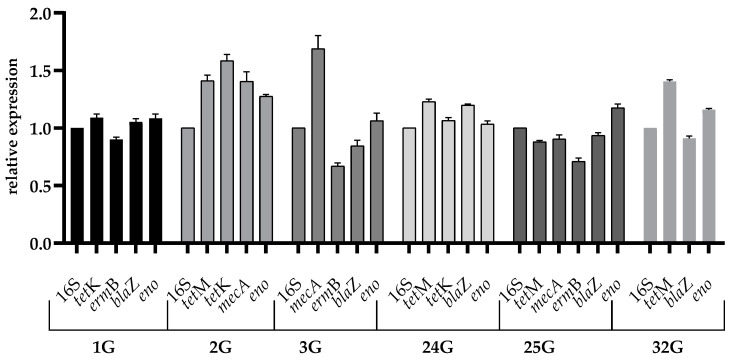
Relative expression of the virulence genes among the tested strains cultured in the sub-inhibitory concentration of cadmium chloride. The error bars represent SD.

Stress induced by SICs of cadmium chloride caused the overexpression of the *eno* gene in all the tested strains belonging to *S. epidermidis* species: 1G (1.05), 2G (1.29), and 3G (1.02) as well as in all the *S. aureus* strains: 24G (1.01), 25G (1.14), and 32G (1.15). Exposure of the tested strains to SICs of cadmium chloride affected overexpression in the following strains: 1G (*S. epidermidis*) *tet*K gene (1.12); 2G (*S. epidermidis*) *tet*M (1.53), *tet*K (1.53) and *mec*A (1.49) genes; 3G (*S. epidermidis*) *mec*A gene (1.77); 24G (*S. aureus*) *tet*M (1.21) and *bla*Z (1.19) genes; and 32G (*S. aureus*) *tet*M gene at 1.39. Reduced expression as a result of sub-inhibitory cadmium chloride stress occurred in strain 1G of the *erm*B gene (0.92), and in strain 3G of the *erm*B (0.69) and *bla*Z (0.81) genes. Strain 25G (*S. aureus*) showed reduced expression of the *tet*M (0.89), *mec*A (0.94), and *erm*B (0.68) genes, while the same was expressed by strain 6G of the *bla*Z gene (0.93). The remaining genes showed expressions similar to that of the reference gene (Figure 2).

## 3. Discussion

The present study aimed to investigate the relationship between biocide tolerance and antibiotic cross-resistance, resistance expression, and virulence. The study showed that after applying SICs of benzalkonium chloride, the MIC values for erythromycin, tetracycline, and oxacillin increased in 83.3%, 66.7%, and 100% of the tested isolates, respectively. Marzoli et al. [12] studied the effect of SICs of benzalkonium chloride on CoNS. They showed that after stress, 16.6% of the isolates became resistant to cefoxitin and intermediate-sensitive to cefotaxime, and 8.3% of *S. epidermidis* were resistant to ampicillin. This finding confirms the impact of SICs of QAC on changes (and even increases) in the antibiotic resistance of the genus *Staphylococcus*.

According to Lawal et al. [14], bacterial exposure to heavy metals has been identified as a selective factor for antibiotic and biocide resistance since the determinants of resistance to these antimicrobials are usually present on the same mobile genetic elements such as plasmids. In staphylococci, the determinants of resistance to heavy metals, including cadmium, are often associated with cassette chromosomes *mec*. Dweba et al. [22] report that the presence of As, Cu, and Zn, even at low levels, increases bacterial resistance to tetracycline. Sinegani et al. [23], studying bacteria isolated from heavy metal-contaminated soils, found that samples from mining waste with higher heavy metal levels had the lowest number of bacteria, but a relatively higher number of bacteria resistant to heavy metals and antibiotics. They also observed a high degree of resistance to ampicillin and amoxicillin in isolates from all soils. In the present study, treatment of strains with an SIC of cadmium chloride contributed to an increase in the MIC for erythromycin in 100% of the tested stains, while for tetracycline and oxacillin in 83.3%. The results obtained in this study confirm that the exposure of bacteria to heavy metals contributes to an increase in their resistance, which may result in the intensification of microbial pathogenicity.

Antibiotic resistance in bacteria is a worldwide problem. Foodborne microorganisms, through horizontal gene transfer (HGT), can acquire drug-resistance genes from resistant cells, and these cells transfer genes through the same pathway to human gastrointestinal bacteria [24]. Sub-inhibitory concentrations of disinfectants in the present study induced changes in the relative expression of the genes responsible for antibiotic resistance (*tet*K*, tet*M, *bla*Z, *erm*B, and *mec*A). Non-inhibitory concentrations of biocides can exacerbate drug-resistant microbial traits. According to the current Commission Regulation (EC) No 2073/2005 of 15 November 2005 on microbiological criteria for foodstuffs, as amended, granules of *Staphylococcus* spp. (excluding enterotoxins produced by *S. aureus* species when the number of these bacteria exceeds 105 cells per gram of product in food) are not included in the hygiene or food safety criteria [25]. The results indicate that both CPS and CoNS possess the genes responsible for antibiotic resistance, and their expression is altered under the influence of SICs of biocides that may be present in the food production pathway. Bacteria present in food that harbor antibiotic resistance genes increase the risk of transferring drug-resistance genes through food consumption or handling itself [25].

Treatment with SICs of benzalkonium chloride overexpressed the *tet*M, *tet*K, *mec*A, and *bla*Z genes in 75%, 66.6%, 33.3%, and 40% of the tested isolates, respectively. Treatment of the isolates with SICs of cadmium chloride overexpressed the same genes in 75%, 100%, 66.6%, and 40% of the strains, respectively. In both cases, the expression of the *erm*B gene was reduced in 100% of the isolates. There are reports that the action of disinfectants at SICs promotes resistance or cross-resistance to other antimicrobial substances [12,26]. In the context of the study, it was observed that increases in the expression levels of antibiotic resistance genes had a noticeable effect on the minimum inhibitory concentration (MIC) values of the antibiotic. However, a decrease in expression did not result in any change in the observed phenotype. Moreover, despite the decrease in *erm*B gene expression, most strains showed an increase in MIC against erythromycin after both stresses. The highest correlation between gene expression and phenotypic resistance was found after cadmium chloride stress for tetracycline resistance with the *tet*M (R^2^ = 0.9134, *p* = 0.1902) and *tet*K (R^2^ = 0.6686, *p* = 0.39) genes and oxacillin resistance with the *mec*A gene (R^2^ = 0.5759, *p* = 0.4515).

The increase in the relative expression values of antibiotic resistance genes is likely related to the action of efflux pumps, which function to remove substances harmful to bacterial cells [27]. The overexpression of efflux pumps and modification of bacterial cell membranes are adaptive mechanisms of microorganisms to disinfectants at SICs [10]. The presence of non-inhibitory concentrations of disinfectants facilitates the spread of mobile genetic elements that simultaneously contain disinfectant and antibiotic resistance genes. Zhang et al. (2017) showed that conjugative transfer within a species in such a case can be faster by up to 7.5 times [28]. There is also evidence that the emergence of so-called cross-resistance may originate precisely in the food industry. The use of QAC and sulfonamides since the 1930s has been linked to the transfer, via food products, of a class 1 integron—a sequence type that plays a major role in the spreading of antibiotic resistance [29,30]. Nowadays, a number of subsequent laboratory studies confirm that exposure of bacteria, including *Staphylococcus* sp., to concentrations of benzalkonium chloride and cadmium ions below the MIC is associated with the occurrence of resistance to the substance and antibiotics, such as tetracyclines, ciprofloxacin, chloramphenicol, polymyxins, ampicillin, and rifampicin [31,32,33].

Biofilm formation makes it easier for foodborne microorganisms to survive in the food production chain. This phenomenon is considered a threat to the food industry. The presence of a bacterial biofilm carries the risk of food contamination with pathogenic microorganisms and food spoilage. The ability of staphylococci to form biofilm is considered the most important process contributing to resistance to harmful external conditions and colonization [34,35]. The present study indicates that changes in phenotypic slime production by the *Staphylococcus* strains occurred as a result of stress induced by SICs of cadmium chloride. However, the present study found no correlation between changes in the *eno* gene expression subsequent to the application of stress factors and the phenotypic changes in slime and biofilm production capacity of the strains that were examined. However, both biocides increased the intensity of biofilm production by the strains analyzed. Other results were obtained by Buzón-Duran et al. [13], who indicated a reduction in the intensity of biofilm production by MRSA following incubation with a SIC of benzalkonium chloride. In contrast, an increase in biofilm production in the cited studies occurred after incubation of the strains in a sub-inhibitory concentration of sodium hypochlorite. This implies the need for further analysis of the effects of non-inhibitory concentrations of disinfectants on microbial characteristics.

## 4. Materials and Methods

### 4.1. Strains

The material for the study comprised 32 strains belonging to the genus *Staphylococcus* spp., including 21 strains of CoNS: *S. epidermidis* (n = 17), *S. haemolyticus* (n = 1), and *S. saprophyticus* (n = 3) and 11 strains CPS: *S. aureus*. All the strains were isolated from dairy products (Table S1) during research conducted at the Department of Food Microbiology, Meat Technology and Chemistry, Faculty of Food Science, University of Warmia and Mazury in Olsztyn. They were previously characterized phenotypically and genotypically using PCR.

### 4.2. Determination of Minimum Inhibitory Concentration of Benzalkonium and Cadmium Chloride

The tested salts of benzalkonium chloride (MP Biomedicals, Illkirch, France) and cadmium chloride (ThermoFisher, Kandel, Germany) were prepared in CA-MHB (Cation-Adjusted Mueller Hinton Broth) (Merck, Darmstadt, Germany) in accordance with Annex C of ISO 20776-1:2019(E) [36] in the concentration range of 0.25–512 μg/mL. The minimum inhibitory concentration (MIC) of the tested compounds was determined using the microdilution method in CA-MHB broth in accordance with ISO 20776. We prepared disinfectants and inoculates of the tested microorganisms with a density of 10^6^ CFU/mL (concentration of inoculates determined based on optical density at 600 nm). Finally, concentrations of the tested compounds were obtained in the range of 0.125–256 μg/mL and the concentration of bacterial inoculates at the level of 5 × 10^5^ CFU/mL. The plates were incubated at 37 °C under aerobic conditions for 18–20 h. The MIC value was read as the lowest concentration of the tested disinfectants in which no bacterial growth was observed (no turbidity of the medium) based on the EUCAST broth microdilution reading guide procedure.

### 4.3. Determination of the Effect of Disinfectants at Sub-Inhibitory Concentrations (SIC) on the Level of Susceptibility of Staphylococci

The tested *Staphylococcus* strains were regenerated by inoculation onto fresh TSB broth (Merck, Darmstadt, Germany). After a 24 h incubation at 37 °C, the strains were passaged onto fresh TSB medium containing benzalkonium chloride and cadmium chloride at the appropriate SIC. The SIC was ½ of the MIC for each strain. Each strain was incubated in 3 replicates. The strains were incubated in the presence of SIC of chloride at 37 °C for a total of 3 days. After each day, the tested strains in the amount of 20 μL were transferred to a fresh medium with the same SIC [12]. The bacteria were then streaked onto TSA agar plates (Merck, Darmstadt, Germany) and incubated for 24 h at 37 °C to check purity. The pure colonies were the material for determining the minimum inhibitory concentrations. MIC testing was performed as described previously.

### 4.4. Effect of SIC on the Virulence and Antibiotic Resistance of Staphylococcus spp.

Analyses were carried out for the 6 chosen strains: *S. aureus* (n = 3) and *S. epidermidis* (n = 3), which possess antibiotic resistance and biofilm-associated genes (Table 4).

#### 4.4.1. Antibiotic Susceptibility Testing by Microdilution Broth Assay

Antibiotic susceptibility profiles for the tested strains were determined by the micro broth dilution method using a 96-well V-bottom polystyrene plate (Promed^®^, Torreglia, Italy) according to ISO 20776-1. In this study, three antibiotics belonging to three different classes were selected: oxacillin, tetracycline, and erythromycin (TOKU-E; Bellingham, WA, USA); they are commonly used in the treatment of *Staphylococcus* infections, including those occurring in animal farming. Antibiotic solution stocks and bacteria suspension were prepared as described in our previous work [37]. The antibiotic susceptibility analysis of the test strains was performed according to CLSI 2020 [38].

#### 4.4.2. Detection of the Ability to Slime Production by Congo Red Agar (CRA) Method

Slime production ability was performed using the Congo Red Assay method as described previously [34]. The plates were incubated for 24 h at 37 °C. Biofilm-forming capacity was interpreted according to colony phenotypes. The appearance of almost black or black colonies was considered as a positive result, while bordeaux and red colonies were considered as negative test results [39]. *S. epidermidis* ATCC 35894 and *S. epidermidis* ATCC 12228 were used as a positive and negative control, respectively.

#### 4.4.3. Determination of Biofilm Production by Microtiter Plate (MTP) Method

The ability of the tested strains to produce biofilm was examined in a 96-well, flat-bottom, sterile polystyrene plate (Promed^®^, Torreglia, Italy) according to the methodology proposed by Stepanovic (2007) [40] as described in our previous paper [34]. Briefly, the absorption at a wavelength of 570 nm was measured using a spectrophotometric microplate reader, the Varioscan LUX (Thermo Scientific, Waltham, MA, USA). Wells containing broth only were used as a negative control. The optical densities (ODs) for each test strain were determined from the arithmetic mean of three replicates, and the value obtained was then compared with the cut-off value (ODc). The ODc was defined as three standard deviations above the mean OD of the negative control. The isolates were then classified according to their OD values as follows: non-biofilm producers (OD ≤ ODc); weak biofilm producers (ODc < OD ≤ 2 × ODc); intermediate biofilm producers (2 × ODc < OD ≤ 4 × ODc); and strong biofilm producers (4 × ODc < OD).

### 4.5. Determination of Expression of Antibiotic Resistance and Virulence Genes

#### 4.5.1. RNA Extraction and Reverse Transcription

Total RNA was isolated using the Total RNA Mini Plus kit (A&A Biotechnology, Gdańsk, Poland), and then purified and concentrated using the Clean-Up RNA Concentrator (A&A Biotechnology, Poland) according to the manufacturer’s instructions. RNA concentration and purity were determined optically using a DeNovix DS-11 FX spectrophotometer/fluorometer (DeNovix Inc., Wilmington, DE, USA) based on the absorbance of the samples at 260 nm and 280 nm. All the RNA samples were partially subjected to immediate reverse transcription, with the remainder stored at −80 °C. Based on the obtained RNA concentration, 5 μg of RNA was transcribed into cDNA using the TranScriba Kit for first-strand cDNA synthesis (A&A Biotechnology, Gdańsk, Poland) [41].

#### 4.5.2. Real-Time PCR Analysis of Gene Expression

Evaluation of the expression of the genes determining virulence was carried out using the primers shown in Table 5. The 16S rRNA gene was used as the housekeeping gene. Expression levels were determined fluorometrically using PowerUp SYBR Green MasterMix (ThermoFischer, Waltham, MA, USA) in a Rotor-Gene^®^ Q thermocycler (Qiagen Inc., Montreal, ON, Canada). Cycling conditions for the reaction for each gene tested were as follows: 50 °C for 2 min, 95 °C for 2 min, 40 cycles of 15 s at 95 °C, followed by 15 s of temperature control for the primers and 60 s at 72 °C [42].

### 4.6. Statistical Analysis

All the statistical analyses were performed by GraphPad Prism 8.0 (GRAPH PAD Software Inc., San Diego, CA, USA). The significance of differences was analyzed at *p* ≤ 0.05.

## 5. Conclusions

Exposure to sub-inhibitory concentrations of disinfectants induces, to varying degrees, changes in antibiotic susceptibility and virulence in both CoNS and CPS. The presence of microorganisms that survive a given stress can pose a serious threat to public health. The results indicate that in *Staphylococcus* spp. strains, changes in the expression of the genes encoding antibiotic resistance and virulence factors can occur under the influence of sub-inhibitory concentrations of cadmium chloride and benzalkonium chloride. The results imply the need for careful selection and adherence to the concentrations of disinfectants and preservatives used to prevent changes at the cellular level.

## Figures and Tables

**Table 1:** MIC values before and after stress induced by sub-inhibitory concentrations of benzalkonium chloride and cadmium chloride.

Species	ID Isolate	MIC Benzalkonium Chloride (µg/mL)		MIC Cadmium Chloride (µg/mL)	
		PRE	POST	PRE	POST
*S. epidermidis*	1G	8	16	8	64
	2G	8	16	4	>128
	3G	4	8	2	8
	4G	2	8	32	256
	5G	4	8	16	>256
	6G	2	4	16	256
	7G	4	16	32	32
	8G	8	8	0.5	128
	9G	4	4	16	16
	10G	16	8	4	256
	11G	4	8	32	8
	12G	4	8	16	128
	13G	2	8	16	64
	14G	8	8	128	64
	15G	4	4	16	>256
	16G	4	8	16	128
	17G	4	8	0.25	128
*S. haemolyticus*	18G	8	16	16	32
*S. saprophyticus*	19G	2	4	32	8
	20G	4	4	128	64
	21G	4	8	16	8
*S. aureus*	22G	4	2	16	32
	23G	4	8	128	>512
	24G	4	8	16	>512
	25G	4	8	128	64
	26G	4	8	128	256
	27G	4	4	256	256
	28G	4	4	64	>256
	29G	4	8	>256	128
	30G	4	4	256	128
	31G	4	8	32	16
	32G	4	16	128	256

**Table 2:** MIC values of selected antibiotics before and after stressing with SICs of benzalkonium chloride (BAC) and cadmium chloride.

ID	Species	MIC (µL/mL) PRE			MIC Values (µL/mL) for Antibiotics After BAC Stress			MIC Values (µL/mL) for Antibiotics After Cadmium Chloride Stress		
		E	TE	OXA	E	TE	OXA	E	TE	OXA
1G	*S. epidermidis*	8	0.25	16	8	0.25	32	32	0.75	16
2G		4	0.25	4	8	0.75	16	8	1	16
3G		2	0.25	0.75	4	1	2	4	1	4
24G	*S. aureus*	0.125	0.125	0.125	0.5	0.125	0.25	0.5	0.125	0.25
25G		1	0.25	2	2	0.5	16	2	0.5	16
32G		1	0.125	0.0625	2	0.25	0.25	2	0.25	0.5

E—erythromycin; TE—tetracycline; OXA—oxacillin.

**Table 3:** Phenotypic changes among tested strains in ability to biofilm formation and slime production.

Species	ID Isolate	Biofilm Formation Ability			Slime Production Ability		
		PRE	POST		PRE	POST	
			Benzalkonium Chloride Treatment	Cadmium Chlroide Treatment		Benzalkonium Chloride Treatment	Cadmium Chlroide Treatment
*S. epidermidis*	1G	intermediate	intermediate	weak	bordeaux	red	bordeaux
	2G	intermediate	strong	strong	bordeaux	bordeaux	bordeaux
	3G	weak	intermediate	strong	bordeaux	red	almost black
*S. aureus*	24G	strong	intermediate	strong	almost black	almost black	almost black
	25G	strong	intermediate	strong	almost black	almost black	almost black
	32G	strong	strong	strong	almost black	almost black	almost black

**Table 4:** Genomic characteristics of chosen strains.

Isolate	Identification	Antibiotic Resistance Genes	Virulence Gene
1G.	*S. epidermidis*	*tet*K, *erm*B, and *bla*Z	*eno*
2G.	*S. epidermidis*	*tet*K, *tet*M, and *mec*A	*eno*
3G.	*S. epidermidis*	*bla*Z, *mec*A, and *erm*B	*eno*
24G.	*S. aureus*	*tet*K, *tet*M, and *bla*Z	*eno*
25G.	*S. aureus*	*mec*A, *tet*M, *erm*B, and *bla*Z	*eno*
32G.	*S. aureus*	*tet*M and *bla*Z	*eno*

**Table 5:** Primers used for Quantitative Real-Time PCR.

Gene	Primers Sequence (5′ → 3′)	Amplicon Size [bp]	References
*Eno* (encodes α-enolase)	F: AAACTGCCGTAGGTGACGAA	301	[43]
	R: TGTTTCAACAGCATCTTCAGTACCTT		
*tet*K (encodes a tetracycline efflux pump)	F: TGCTGCATTCCCTTCACTGA	69	[44]
	R: GCTTTGCCTTGTTTTTTTCTTGTAA		
*tet*M (ribosomal protection protein)	F: CAGAATTAGGAAGCGTGGACAA	67	
	R: CCTCTCTGACGTTCTAAAAGCGTAT		
*erm*B (encodes the ribosomal methylase)	F: GGATTCTACAAGCGTACCTTGGA	69	
	R: AATCGAGACTTGAGTGTGCAAGAG		
*mec*A (encodes penicillin-binding protein 2a)	F: CAATGCCAAAATCTCAGGTAAAGTG	107	[45]
	R: AACCATCGTTACGGATTGCTTC		
*bla*Z (encoding penicillin resistance)	F: GCTTTAAAAGAACTTATTGAGGCTTCA	233	
	R: CCACCGATYTCKTTTATAATTT		
16s rRNA	F: CCGCCTGGGGAGTACG	240	
	R: AAGGGTTGCGCTCGTTGC		

## Data Availability

Data are contained within the article and Supplementary Materials.

## Supplementary Materials

The following supporting information can be downloaded at https://www.mdpi.com/article/10.3390/ijms26062429/s1.
