# Supplementary material for: Effect of Sub-Inhibitory Concentrations of Quaternary Ammonium Compounds and Heavy Metals on Antibiotic Resistance and Expression of Virulence Factors Among Staphylococcus spp. from Dairy Products
Source: Int J Mol Sci. 2025 Mar 8;26(6):2429. doi: 10.3390/ijms26062429 (PMC11941806; doi:10.3390/ijms26062429)
Supplement: Supplementary file 1 [file ijms-26-02429-s001.zip › ijms-3479556-supplementary.pdf]

Supplement Table S1. Identification and characteristic of strains isolated from dairy products.

ID	Identification MALDI-TOF	Source	Antibiotic resistance genes	Biofilm forming associated genes
**1G**	*S. epidermidis*	cheese	*bla*Z, *tet*K, *erm*B	*eno*
**2G**		raw cow's milk	*mec*A, *tet*K, *tet*M	*eno*
**3G**		raw cow's milk	*bla*Z, *mec*A, *erm*B	*eno*
**4G**		cheese	*bla*Z, *tet*K	*eno*
**5G**		cheese	*bla*Z	*eno*
**6G**		cheese	*bla*Z, *tet*K, *tet*L, *erm*B	*eno*
**7G**		whey	*bla*Z, *erm*B	*eno*
**8G**		cheese	*bla*Z, *erm*B	*eno*
**9G**		raw cow's milk	*bla*Z, *erm*B	*eno*
**10G**		cream	*bla*Z, *mec*A, *erm*B	*eno*
**11G**		raw cow's milk	*bla*Z, *mec*A, *tet*L	*eno*
**12G**		raw cow's milk	*bla*Z, *mec*A, *erm*B	*eno*
**13G**		cream	*bla*Z, *mec*A	*eno*
**14G**		cheese	*bla*Z, *mec*A, *tet*K, *erm*B	*eno*
**15G**		cheese	-	*eno*
**16G**		cheese	*mec*A	*eno*
**17G**		cheese	*mec*A	*eno*
**18G**	*S. haemolyticus*	raw cow's milk	*bla*Z	*eno*
**19G**	*S. saprophyticus*	cheese	*tet*K, *tet*M	*eno*
**20G**		cheese	*tet*M	*eno*
**21G**		cheese	*bla*Z, *tet*K, *tet*M	*eno*
**22G**	*S. aureus*	raw cow's milk	*bla*Z, *tet*M, *erm*B,	*eno*
**23G**		cheese	*bla*Z, *tet*M	*eno*
**24G**		whey	*bla*Z, *tet*K, *tet*M	*eno*
**25G**		cheese	*bla*Z, *mec*A, *tet*M, *erm*B	*eno*
**26G**		raw cow's milk	*bla*Z	*eno*
**27G**		raw cow's milk	*bla*Z, *mec*A, *tet*M, *erm*B	*eno*
**28G**		cheese	*bla*Z, *tet*M, *erm*B	*eno*
**29G**		cheese	*bla*Z	*eno*
**30G**		raw cow's milk	*bla*Z, *tet*M	*eno*
**31G**		cheese	*bla*Z, *tet*M, *erm*B	*eno*
**32G**		raw cow's milk	*bla*Z, *tet*M	*eno*
